# A Retrospective Study on Clinical Outcomes of Pregnancy-Related Acute Kidney Injury Patients at a South Indian Tertiary Care Hospital

**DOI:** 10.7759/cureus.49610

**Published:** 2023-11-28

**Authors:** Maniyar Iqbal Anvar, Sidhant Talwar, Shashikanth Mallapur

**Affiliations:** 1 Nephrology, Vijayanagar Institute of Medical Sciences (VIMS), Bellary, IND; 2 Internal Medicine, Vijayanagar Institute of Medical Sciences (VIMS), Bellary, IND

**Keywords:** perinatal mortality, hemodialysis, sepsis, stage of aki, pregnancy-related aki

## Abstract

Introduction

Acute kidney injury (AKI) significantly contributes to maternal morbidity and mortality in developing nations. In a retrospective study conducted at our tertiary care center in collaboration between the Department of Nephrology and the Department of Obstetrics and Gynecology, we investigated patients admitted with pregnancy-related acute kidney injury (PR-AKI) under the following parameters: incidence, etiology, and maternal outcomes.

Methods

We evaluated 70 patients admitted with PR-AKI from May 2016 to August 2020. A thorough evaluation was carried out for these patients. The results were analyzed for the association of mortality with the etiology of PR-AKI and the dialysis requirement.

Results

The mean age among the PR-AKI patients was 24.56 ± 4.2 years. During the study period, there were 33,403 deliveries, consisting of 20,126 vaginal deliveries, and 13,277 were performed via a lower segment cesarean section (LSCS). Seventy patients developed AKI, with an incidence of 2.9 per 1,000 deliveries. The various etiologies included sepsis in 54 cases (74.3%), preeclampsia/eclampsia in 44 (62.85%), LSCS in 27 (38.6%), abruptio placentae in 11 (15.7%), postpartum hemorrhage (PPH) in 11 (15.7%), post-abortion in eight (11.4%), and hemolysis, elevated liver enzymes, and low platelets (HELLP) syndrome in seven (10.46%). The number of patients in various stages of AKI was noted as one in Stage I, 22 in Stage II, and 47 in Stage III. The odds ratio of death in the abruptio placentae was 0.73 (95% CI: 0.08-6.72), whereas among those with PPH, it was 1.96 (95% CI: 0.34-11.29). The odds ratio of death among patients with LSCS was 0.50 (95% CI: 0.09-2.64). Out of the total, 34 patients (48.6%) required renal replacement therapy (RRT) provided as intermittent hemodialysis. In total, there were eight deaths (11.3%). The odds ratio of death in dialysis patients was 1.89 (95% CI: 0.42-8.54). Perinatal mortality was 32.9%, whereas total perinatal mortality among all patients was 3.5%. The odds ratio of perinatal mortality among those with AKI was 13.29 (95% CI: 8.05-21.96) with p < 0.0001.

Conclusion

Our study demonstrates that sepsis was the most common cause of PR-AKI, which can be attributed to a lack of antenatal and postnatal care. Other causes included preeclampsia, LSCS, and hemorrhage. The present study also shows that a significant association exists between PR-AKI and perinatal mortality. The requirement of RRT in AKI predicts a less favorable prognosis.

## Introduction

Acute kidney injury (AKI) may occur due to obstetric complications such as septic abortion, abruptio placentae, uterine hemorrhage, intrauterine fetal death, and sepsis in previously healthy kidneys. Significant disparities persist in the epidemiological variables of pregnancy-related acute kidney injury (PR-AKI), encompassing incidence, etiology, and outcomes between developed and developing countries, mainly due to socioeconomic and environmental factors [1-3]. With the liberalization of abortion laws and improved obstetrics care in developed countries, PR-AKI is now uncommon [4,5]. A study by Stratta et al. showed that from 1958 to 1994, the incidence of PR-AKI decreased from 1/3,000 pregnancies to 1/18,000 (from 43% to 0.5%) [4]. In developing countries, however, the PR-AKI incidence has declined over the last three decades but still constitutes 5%-20% of total AKI [6-8].

PR-AKI is commonly associated with hypertensive pregnancy disorders, chronic kidney disease (CKD), and hypertension, which correlate to an increased risk of cardiovascular disease later in life [9-11]. The maternal mortality rate in developing countries was 239/100,000 females compared to 12/100,000 in developed countries, thus signifying a higher disease burden [12]. Nonetheless, a retrospective study conducted in the United States in 2016 demonstrated an annual increase of 10% in PR-AKI between the periods 1999-2001 and 2010-2011 (95% CI: 8-11) [13]. Interestingly, despite this increase in incidence, an overall decrease in the severity of AKI was observed. However, it was noteworthy that there was an increase in AKI-associated maternal mortality and dialysis treatment during the same period [13]. Factors that have been hypothesized to contribute to the rise in PR-AKI include increasing pregnancies among females of advanced maternal age (35 years or older), obesity, diabetes, hypertension, multifetal gestation, lower segment cesarean section (LSCS), induction of labor, polyhydramnios, antepartum hemorrhage, cardiac failure, lupus erythematosus, and CKD [13,14].

## Materials and methods

Study design and participants

This retrospective study was conducted at the Department of Nephrology in association with the Department of Obstetrics and Gynecology at Vijayanagar Institute of Medical Sciences, Bellary, India, from May 2016 to August 2020. The study protocol was approved by the Institutional Ethics Committee (letter number: VIMS/STD/PG/SYN/06/2020-2021).

We collected data from all patients admitted with PR-AKI, defining AKI according to the serum creatinine criteria of Kidney Disease: Improving Global Outcomes (KDIGO) guidelines. Pregnant females who had preexisting CKD were excluded from the study.

Data collection

We gathered data on demographic details, clinical presentations, duration of stay, obstetric background, history of hypertension/LSCS/antepartum hemorrhage/postpartum hemorrhage (PPH)/preeclampsia or eclampsia, laboratory parameters, and treatment provided. We also collected data on the requirement for renal replacement therapy (RRT) provided by intermittent hemodialysis and its outcomes, such as death or discharge. Additionally, details of the total number of normal deliveries, LSCS, and perinatal and maternal mortality data were collected during the study period and entered into an Excel (Microsoft® Corp., Redmond, WA) spreadsheet.

Definitions

AKI was defined by the serum creatinine criteria of the KDIGO guidelines, which state it as an absolute increase in serum creatinine of at least 0.3 mg/dL (26.5 μmol/L) within 48 hours or by a 50% increase in serum creatinine from baseline within seven days [15]. PR-AKI was defined as AKI due to any cause related to pregnancy from conception to six weeks postpartum. Postpartum AKI was defined as AKI diagnosed from childbirth to six weeks post delivery. Preeclampsia was defined as a blood pressure reading of >140/90 mmHg diagnosed for the first time after 20 weeks of gestation with 2+ proteinuria on a dipstick. Eclampsia was defined as a new onset of grand mal seizures in females with preeclampsia [16]. Hemolysis, elevated liver enzymes, and low platelets (HELLP) syndrome was defined by a combination of thrombocytopenia (<100,000 × 10^9^/L), elevated liver enzymes (aspartate transaminase {AST} > 70 U/L), and hemolysis. Sepsis was defined as a clinical syndrome that occurs due to a dysregulated inflammatory response to an infection, as per the criteria laid down by the American College of Chest Physicians [17]. Puerperal sepsis was defined as any bacterial infection in the genital tract that occurs after the birth of the baby [18]. PPH was defined as a blood loss of ≥500 mL after vaginal delivery or ≥1000 mL after LSCS or as noted in the medical records as per the care provider [18].

Outcomes

We noted patient outcomes for mortality, duration of stay, requirement of RRT, and recovery of renal function (dialysis independence, decreasing serum creatinine to <1.0 mg/dL, or increased urine output). Pregnancy outcomes such as mortality, preterm delivery, and abortion were also recorded.

Statistical analysis

The data was analyzed using the Statistical Package for Social Sciences (SPSS) software version 20.0 (IBM SPSS Statistics, Armonk, NY).

## Results

During the study period, there were 33,403 deliveries, of which 20,126 were normal vaginal deliveries and 13,277 were performed by LSCS, resulting in a section rate of 39.75%. Out of the total deliveries, 70 patients developed PR-AKI. Within this group of 70 cases, only one was attributed to a ruptured ectopic pregnancy, while the remaining 69 patients had postpartum AKI. The incidence of PR-AKI in our study was 2.09 per 1,000 deliveries. The mean age of our patients was 24.56 ± 4.2 years.

The total maternal mortalities during the study period were 138 females out of 33,403 deliveries, equivalent to a rate of 4.31 per 1,000 deliveries. Eight of the 70 PR-AKI patients expired (11.42%), which equates to 114 deaths per 1,000 AKI patients, highlighting an increased mortality rate in this group.

Perinatal mortality was seen in 23 cases (32.9%) out of the 70 PR-AKI patients, while total perinatal mortality among all 33,403 patients during the study period was 3.5%, accounting for 1,186 cases. The odds ratio of perinatal mortality among those with AKI was 13.29 (95% CI: 8.05-21.96) with p < 0.0001.

**Figure 1:**
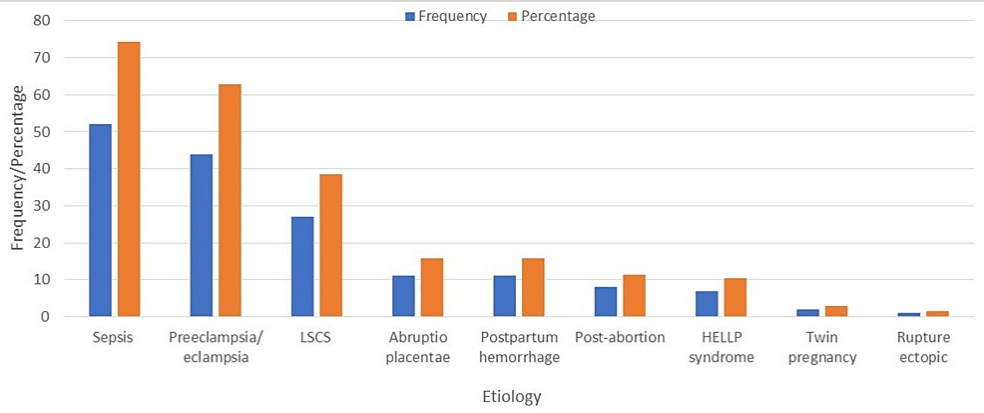
Distribution of etiology in pregnancy-related acute kidney injury cases LSCS, lower segment cesarean section; HELLP, hemolysis, elevated liver enzymes, and low platelets

Among the 70 patients who developed AKI, sepsis was the most common etiology, present in 52 patients (74.3%), followed by preeclampsia/eclampsia, responsible for AKI in 44 patients (62.85%). LSCS was the etiology in 27 patients (38.6%); thus, among 13,277 LSCS patients, the incidence of AKI was 2.03 per 1,000 LSCS. Other causes included abruptio placentae and postpartum hemorrhage, both of which were present in 11 patients (15.7%) each (Table 1 and Figure 1).

**Table 1:** Distribution of etiology in pregnancy-related acute kidney injury LSCS, lower segment cesarean section; HELLP, hemolysis, elevated Liver enzymes, and low platelets

Etiology	Frequency	Percentage (%)
Sepsis	52	74.33
Preeclampsia/eclampsia	44	62.85
LSCS	27	38.6
Abruptio placentae	11	15.7
Postpartum hemorrhage	11	15.7
Post-abortion	8	11.4
HELLP syndrome	7	10.46
Twin pregnancy	2	2.8
Rupture ectopic	1	1.4

Among those who developed AKI, 25 females (35.5%) had antenatal monitoring throughout pregnancy, while 45 females (64.5%) did not and were seen for the first time during the delivery (Table 2). Additionally, 40 patients (57%) out of 70 delivered at full term, while 30 (43%) were preterm. Common symptoms included pedal edema in 52 patients (74.2%), followed by oliguria in 47 patients (67.1%), dyspnea in 46 patients (65%), and orthopnea in 23 patients (32.9%) (Table 3). The number of patients in various stages of AKI was noted as one in Stage I, 22 in Stage II, and 47 in Stage III (Table 4).

**Table 2:** Description of antenatal monitoring in pregnancy-related acute kidney injury cases

Antenatal monitoring	Number of patients	Percentage (%)
Present	25	35.5
Absent	45	64.5

**Table 3:** Distribution of symptoms in pregnancy-related acute kidney injury cases

Symptoms	Frequency	Percentage (%)
Pedal edema	52	74.2
Oliguria	47	67.1
Exertional dyspnea	46	65
Fever	42	60
Orthopnea	23	32.9
Vomiting	19	27.1
Anuria	18	25.7
Jaundice	14	20
Cough	6	8.6
Seizure	3	4.3

**Table 4:** Distribution of various stages of acute kidney injury among the study population

Stage of acute kidney injury	Number of patients	Percentage (%)
I	1	1.43
II	22	31.43
III	47	67.14

**Figure 2:**
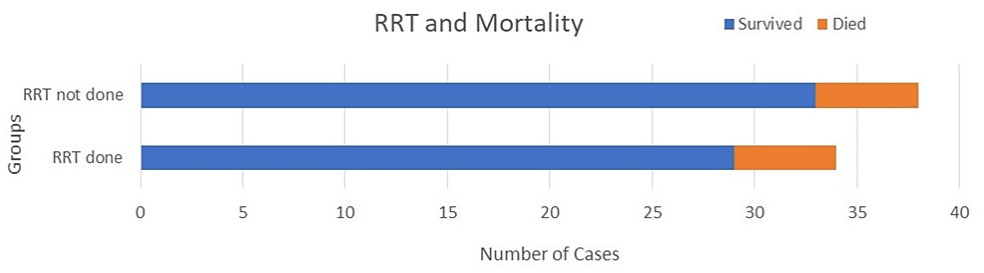
Comparison of survival and mortality in patients who underwent renal replacement therapy (RRT)

Out of the 70 patients, 34 (48.6%) required RRT, which was provided through intermittent hemodialysis. Among these 34 patients, five expired (14.1%), and 29 successfully recovered (85.9%). For the remaining 36 patients who did not require RRT, three expired (8.4%), and 33 survived (91.6%) (Table 5 and Figure 2). The odds ratio of death for patients who required RRT was 1.89 (95% CI: 0.92-8.54) with p < 0.472.

**Table 5:** Comparison of survival and mortality in patients who underwent renal replacement therapy (RRT)

RRT	Number of patients (%)	Survived (%)	Died (%)
Done	34 (48.6%)	29 (85.9%)	5 (14.1%)
Not done	36 (51.4%)	33 (91.6%)	3 (8.4%)

A total of 11 PPH patients had AKI, of which two expired (18.2%) and nine survived (81.8%). Among the other 59 patients with no PPH, six expired (10.2%), and 53 survived (89.8%) (Table 6). The odds ratio of death due to PPH causing AKI was 1.96 (95% CI: 0.34-11.29) with p < 0.456.

**Table 6:** Outcome comparison for patients with pregnancy-related acute kidney injury (PR-AKI) with and without postpartum hemorrhage (PPH)

Disease	Number of patients (%)	Survived (%)	Died (%)
PR-AKI with PPH	11 (15.7%)	9 (81.8%)	2 (18.2%)
PR-AKI without PPH	59 (84.3%)	53 (89.8%)	6 (10.2%)

Among the 11 cases of abruptio placentae, one patient (9.5%) expired, while 10 survived (90.5%). In contrast, among the 59 patients without abruption, seven expired (11.9%), and 52 survived (88.1%) (Table 7). The odds ratio of death among these patients was 0.743 (95% CI: 0.08-6.72) with p < 0.791.

**Table 7:** Comparison of patient outcomes in cases of pregnancy-related acute kidney injury (PR-AKI) with and without placental abruption

Disease	Number of patients (%)	Survived (%)	Died (%)
PR-AKI with abruption	11 (15.7%)	10 (90.5%)	1 (9.5%)
PR-AKI without abruption	59 (84.3%)	52 (88.1%)	7 (11.9%)

Out of the 27 patients who had AKI following a lower segment cesarean section (LSCS), two patients died (7.4%), while 25 patients survived (92.6%). However, among the 43 patients who developed AKI after vaginal delivery, six died (14%), and 37 survived (86%) (Table 8). The odds ratio of death among those with LSCS as the etiology of AKI was 0.50 (95% CI: 0.09-2.64) with p < 0.334. A summary of various parameters and odds ratios is given in Table 9.

**Table 8:** Comparison of outcomes of pregnancy-related acute kidney injury (PR-AKI) in cesarean section and vaginal delivery cases LSCS: lower segment cesarean section

Disease	Number of patients (%)	Survived (%)	Died (%)
PR-AKI in LSCS	27 (38.6%)	25 (92.6%)	2 (7.4%)
PR-AKI in vaginal delivery	43 (61.4%)	37 (86%)	6 ( 14%)

**Table 9:** Association of various parameters with mortality in pregnancy-related acute kidney injury patients LSCS, lower segment cesarean section; PPH, postpartum hemorrhage

Variable		Death		Total	OR (95% CI)	Chi-square	P value
		No	Yes				
LSCS	No	37 (86.0%)	6 (14.0%)	43 (100%)	0.50 (0.09-2.64)	0.702	0.334
	Yes	25 (92.6%)	2 (7.4%)	27 (100%)			
Abruptio placentae	No	52 (88.1%)	7 (11.9%)	59 (100%)	0.743 (0.08-6.72)	0.070	0.791
	Yes	10 (90.9%)	1 (9.1%)	11 (100%)			
Dialysis	No	33 (91.7%)	3 (8.3%)	36 (100%)	1.89 (0.42-8.64)	0.701	0.472
	Yes	29 (85.3%)	5 (14.7%)	34 (100%)			
PPH	No	53 (89.8%)	6 (10.2%)	49 (100%)	1.96 (0.34-11.29)	0.555	0.456
	Yes	9 (81.8%)	2 (18.2%)	11 (100%)			

The duration of hospital stays among those who survived and those who expired was also analyzed. The mean duration of stay of patients who passed away was 5.5 days (range: 5-10.5), while the same for those who survived was 9.5 days (range: 7-15.5). The patients who died had more severe anemia, sepsis, and transaminitis, but this was statistically insignificant. The distribution of the incidence of PR-AKI with various parameters has been shown in Table 10.

**Table 10:** Comparison of various parameters among the total study population to PR-AKI patients PR-AKI, pregnancy-related acute kidney injury; HELLP, hemolysis, elevated liver enzymes, and low platelets

Parameters	Total cases	PR-AKI
Total deliveries	33,403	70
Vaginal delivery	20,126	43
Cesarean section	13,277	27
Breech	615	1
Twins	375	2
Fetal death	816	23
Abruptio placentae	284	11
Preeclampsia/eclampsia	6,662	44
HELLP syndrome	86	9
Maternal mortality	138	8
Perinatal mortality	1,186	23

## Discussion

The incidence of PR-AKI is significantly higher in the developing world than in developed countries. In two studies of developed countries, the incidence of PR-AKI was 0.1% and 0.5% of the total AKI [4,5]. Stratta et al. conducted a study examining the incidence of PR-AKI in Italy over several decades, revealing that the incidence had reduced from 1/3,000 to 1/18,000 of total pregnancies between 1956-1967 and 1988-1994 [4]. This contrasted significantly with our current study, where the incidence of AKI was much higher at 2.09 per 1,000 deliveries.

In our series, 69 of the 70 total AKI cases had postpartum AKI (98.5%). In contrast, studies done by Arora et al. [19] and Hassan et al. [20] revealed that the incidence of postpartum AKI contributed to 70%-83% of PR-AKI cases.

The higher incidence of AKI is largely a result of a lack of antenatal and postnatal care, which is reflected in the fact that the most common etiology is sepsis. In our study, 52 patients (74.3%) had sepsis, aligning with the results of the study done by Eswarappa et al. [18], where sepsis was the cause in 75% of cases. Arora et al. likewise reported a similar finding, showing that sepsis was the leading cause of maternal mortality (accounting for 18%-30% of cases), with an incidence of 33.3% [19]. Although all 70 patients had institutional delivery, only 35.5% in our study had antenatal monitoring, and 64.5% had no antenatal follow-up, reflecting a lack of antenatal care, which remains a significant challenge in many low-income countries, including India [21].

Huang and Chen demonstrated that preeclampsia/eclampsia and PPH were the leading causes of PR-AKI in China [22]. In their study, 17% of females diagnosed with preeclampsia/eclampsia also had AKI. In contrast, in our research, out of 6,662 patients who had preeclampsia/eclampsia, 44 patients developed AKI, resulting in an incidence of 0.66%. This difference in incidence can be attributed to our utilization of KDIGO criteria to diagnose AKI, whereas in the above study, they used serum creatinine of >0.70 mmol/L as AKI criteria [22].

In the current study, among 86 patients with HELLP syndrome, seven patients developed AKI, with an incidence of 10.46%. These findings were similar to the 20% incidence reported by Martínez de Ita et al. [23] and were in contrast to the results by Huang and Chen [22], which had a 60% incidence of AKI among HELLP syndrome patients. This difference in the incidence of PR-AKI was because Huang and Chen used a lower serum creatinine criteria to define AKI; however, when they used serum creatinine of >106.8 mmol/L, the incidence of AKI in HELLP syndrome decreased to 13 cases (24.5%) [22].

In previous decades, septic abortion had been the most common cause of AKI during pregnancy and was a significant public health problem in developing countries [2]. A study by Najar et al. reported that septic abortion accounted for 20 cases (50%) of all PR-AKI cases, 15 (75%) of which were in the first trimester and five (25%) were in the second trimester [24]. In contrast, in our present study, there were no cases of AKI due to septic abortion, and only one patient (1.4%) had ruptured ectopic pregnancy as a cause of AKI. We had 11 patients (15.7%) who developed AKI due to PPH, which was less when compared to the study done by Bokhari et al., where 17 out of 41 patients (41%) had AKI due to PPH [25]. Among the 70 PR-AKI patients, three (4.2%) were diagnosed with acute cortical necrosis on renal biopsy, which was done due to slow and partial recovery.

In our study, perinatal mortality was seen in 23 cases (32.9%) out of the 70 PR-AKI patients, whereas total perinatal mortality among the 33,403 patients during the study period was 3.5%, which amounted to 1,186 cases. The odds ratio of perinatal mortality among those with AKI was 13.29 (95% CI: 8.05-21.96) with p < 0.0001. A systemic review and meta-analysis conducted by Liu et al. revealed a substantially lower odds ratio for perinatal death, calculated at 3.39 (95% CI: 2.76-4.18) [26].

The number of females who received dialysis in our study was 34 out of 70, with five deaths among them. The odds ratio of death among those dialyzed was 1.89 (95% CI: 0.42-8.64) with p < 0.472. This result was similar to a study conducted by Hildebrand et al., where they reviewed 1,918,789 pregnancies [5]. In their study, AKI complicated 188 cases, of which eight females died (4.3% versus 0.01% in the general population) [5]. In contrast, Huang and Chen showed that out of 14 patients with PR-AKI requiring RRT, seven died [22]. These findings emphasize that severe AKI is a strong predictor of poor response and outcome.

Limitations

This study had several limitations, including the following: 1) This study has a limited sample size of cases; 2) it was a retrospective study, so it was reliant on medical records and resulted in difficulty in establishing causal relationships; 3) it was a single-center study, so it may not fully represent the entire population; and 4) this study has selection bias: patients with mild PR-AKI who did come to our institute were not included in the analysis.

## Conclusions

AKI during the antenatal and postnatal periods is not rare. In our study, the incidence of PR-AKI was 2.09 per 1,000 deliveries. Sepsis was the most common cause (74.3%), followed by preeclampsia/eclampsia in 44 cases (62.8%) and LSCS in 27 cases (38.6%). AKI was common among those without antenatal monitoring during pregnancy (64.5%). AKI needing dialysis might predict poor outcomes. The perinatal mortality among those with AKI was statistically significant. Thus, increased awareness and improved antenatal and postnatal monitoring are essential to mitigating the occurrence of PR-AKI and its complications.
